# Current status and trends of immune-related adverse events in lung cancer treated with immune checkpoint inhibitors: a bibliometric analysis of the past decade (2016–2025)

**DOI:** 10.3389/fimmu.2026.1846212

**Published:** 2026-06-02

**Authors:** Bing Guo, Ge Zhang, Shengpeng Sang, Xianfen Ma, Huanpeng Qi

**Affiliations:** 1 Department of Comprehensive Oncology, The Affiliated Taian City Central Hospital of Qingdao University, Taian, China; 2 Department of Minimally Invasive Oncology, The Affiliated Taian City Central Hospital of Qingdao University, Taian, China; 3 Department of Clinical Laboratory, The Affiliated Taian City Central Hospital of Qingdao University, Taian, China

**Keywords:** bibliometric analysis, bibliometrix, citespace, immune checkpoint inhibitors, immune-related adverse events, lung cancer

## Abstract

**Background:**

Immune checkpoint inhibitors (ICIs) are widely used in lung cancer treatment; however, immune-related adverse events (irAEs) remain an important safety-related research topic. Currently, there is a lack of comprehensive bibliometric analyses specifically addressing irAEs in lung cancer.

**Methods:**

This study utilized the Web of Science Core Collection (WoSCC) and PubMed databases to retrieve literature on irAEs associated with ICIs in lung cancer from 2016 to 2025. Among the 1, 792 eligible records from WoSCC, bibliometric and visualization analyses were conducted using tools such as Bibliometrix and CiteSpace (v7.0). Meanwhile, PubMed records classified as clinical studies were used only for supplementary descriptive clinical-topic mapping, focusing on study types, disease settings, intervention regimens, and irAE-related topic categories.

**Results:**

The results indicate that the annual publication volume in this field showed a continuous upward trend from 2016 to 2025, progressing through three distinct phases: the initial phase (2016–2019), the rapid increase phase (2020–2022), and the stable development phase (2023–2025);China ranked first globally with 626 publications, while the United States showed the highest betweenness betweenness centrality in the international collaboration network, suggesting a relatively strong bridging position; however, China’s high publication output was not accompanied by a similarly high network betweenness centrality; co-citation analysis identified two major groups of frequently co-cited references: landmark randomized trials of ICIs in lung cancer and guidelines or reviews on irAE management; Research hotspots exhibit a four-stage migration pattern: “originating from pan-tumor safety exploration in melanoma—focusing on lung cancer-specific high-risk irAEs—extending to efficacy-safety association analyses—and expanding to long-term prognosis in recurrent populations.” Keyword burst analysis suggested increasing recent attention to recurrent disease, progression-free survival, and selected safety-related topics. The PubMed-based supplementary mapping further showed that the included clinical studies mainly involved combination immunotherapy, pulmonary toxicity, perioperative treatment settings, special populations, biomarker-related analyses, and irAE intervention-related topics.

**Conclusion:**

This bibliometric analysis describes the publication landscape, collaboration patterns, co-citation structure, and keyword evolution of ICI-related irAE research in lung cancer. The findings may help researchers identify major topic distributions and areas requiring further investigation, particularly organ-specific high-risk irAEs, biomarker-related studies, and safety-focused research in underrepresented populations.

## 1 Introduction

Lung cancer is the most common malignant tumor in terms of both incidence and mortality worldwide. According to data from the 2020 Global Cancer Statistics Report, there are approximately 2.2 million new cases of lung cancer and 1.8 million deaths annually worldwide, accounting for 11.4% and 18.0% of all new cases and deaths from malignant tumors, respectively, placing a heavy burden on global public health systems (1).In China, lung cancer is also the leading cause of cancer-related deaths. In 2016, there were approximately 828, 000 new cases of lung cancer and 657, 000 deaths in China, with incidence and mortality rates continuing to rise year by year (2).

A series of landmark clinical trials, including KEYNOTE-024, KEYNOTE-189, and PACIFIC, have successively demonstrated significant survival benefits of PD-1/PD-L1 inhibitors in first-line treatment of non-small cell lung cancer (NSCLC), as well as in combination with chemotherapy and as consolidation therapy following concurrent chemoradiotherapy, establishing ICIs as the standard of care throughout the entire course of lung cancer treatment (3–5). As of 2025, ICIs have been approved for the treatment of nearly all pathological subtypes of lung cancer, including NSCLC and small cell lung cancer, with their clinical applications continuing to expand.

However, while ICIs provide significant survival benefits, they can also induce abnormal immune activation in the body, leading to irAEs involving multiple organs (6). Unlike the adverse reactions associated with traditional chemoradiotherapy and targeted therapy, irAEs can affect nearly all organ systems throughout the body. Among these, high-grade irAEs such as immune-related pneumonia, immune-related myocarditis, and immune-related hepatitis are potentially fatal and represent major causes of treatment discontinuation and patient mortality (7). Due to impaired baseline lung function and multiple comorbidities, lung cancer patients face a significantly higher risk of developing irAEs and a higher mortality rate following ICI therapy compared to patients with other cancer types. The incidence of immune-related pneumonia can reach 3%–5%, with a mortality rate exceeding 30%, making it the most critical clinical challenge in the comprehensive management of ICI therapy for lung cancer (8, 9).Currently, the American Society of Clinical Oncology (ASCO), the European Society for Medical Oncology (ESMO), and the Society for Immunotherapy of Cancer (SITC) have all published guidelines on irAEs management (10–12); however, there remains a significant unmet clinical need for risk prediction, early identification, and personalized intervention strategies specific to irAEs in the lung cancer population.

In recent years, the volume of clinical and basic research on ICI-related irAEs in lung cancer has exploded; however, existing studies have largely focused on single-center analyses of clinical characteristics and small-sample explorations of mechanisms, lacking a comprehensive, systematic overview of the global research landscape, developmental trajectory, key topics, and cutting-edge trends in this field. Bibliometrics is an interdisciplinary field combining mathematics, statistics, and bibliography. Through visualization techniques, it enables quantitative analysis of publication characteristics, collaboration networks, co-citation relationships, and topic clustering across vast amounts of literature, thereby accurately identifying research hotspots and cutting-edge trends in a given field. It has been widely applied to assess the current state of research in the medical field (13). Although a small number of bibliometric studies on ICI-related irAEs have been conducted, most focus on a broad range of tumor types. There remains a significant lack of bibliometric analyses specifically targeting lung cancer-specific irAEs during the comprehensive application phase of ICIs.

Therefore, this study utilizes the Web of Science Core Collection database and employs bibliometric methods to systematically analyze global literature on lung cancer ICI-related irAEs from 2016 to 2025, mapping publication trends, key contributing countries/institutions/authors, international collaboration networks, core knowledge bases, research hotspots, and emerging trends in this field. In addition, PubMed clinical-study records were used as a supplementary source for descriptive clinical-topic mapping. This component was intended only to provide an overview of the types of clinical studies and topic categories represented in PubMed, rather than to synthesize clinical evidence or formulate practice recommendations.

## 2 Materials and methods

### 2.1 Search strategy

We conducted a literature search and collected articles from the WOSCC and PubMed databases. We used controlled vocabulary terms and free-text terms related to “immune checkpoint inhibitors, ” “immune-related adverse events, ” and “lung neoplasms”; the specific search query is provided in **Supplementary Material 1**. For the search of the WOSCC database, we used topic-based retrieval to obtain more comprehensive data. For the PubMed database, we conducted the search using both Medical Subject Headings (MeSH) terms and free keywords. The search for free keywords was limited to the title/abstract section, which has enabled us to obtain more accurate data.

The WoSCC dataset was used as the primary source for bibliometric and visualization analyses. PubMed was used only for supplementary descriptive clinical-topic mapping to summarize the distribution of clinical study types, disease settings, intervention regimens, and irAE-related topic categories among the included clinical studies. No comparative efficacy or safety conclusions were drawn from the PubMed dataset, and the PubMed analysis was not used to generate clinical recommendations.

### 2.2 Inclusion criteria

We included publications in WOSCC that reported adverse events associated with ICI therapy for lung cancer, limiting the search to English-language articles and reviews. For publications in the PubMed database, we included only those classified as clinical studies.

### 2.3 Exclusion criteria

Literature that did not meet the inclusion criteria was excluded, such as guidelines, conference proceedings, news articles, and letters. Literature under topics unrelated to medicine was also excluded.

### 2.4 Literature screening process

**Figure 1:**
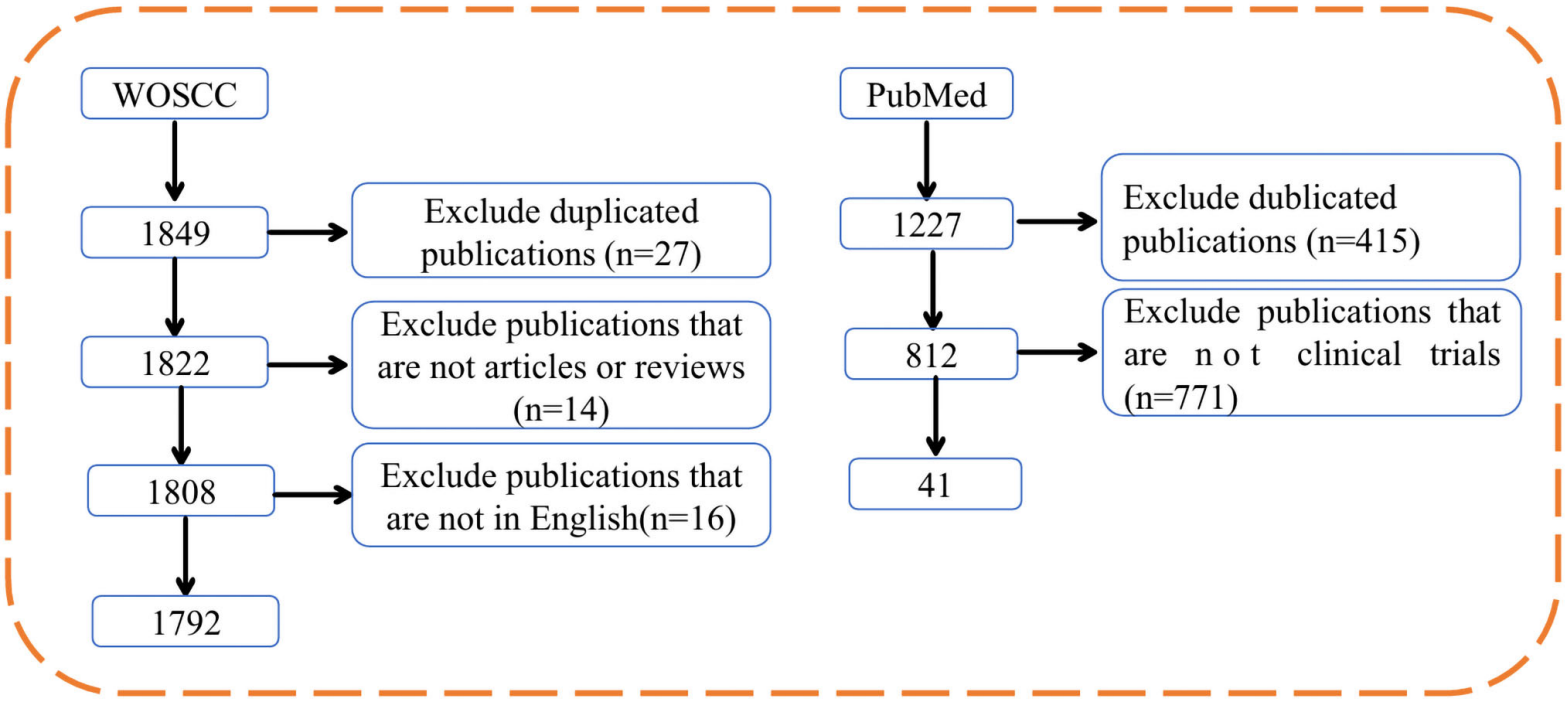
Screening process for included publications.

Based on the preliminary search results, we first used EndNote X9 software to remove duplicates, then screened the results according to the inclusion and exclusion criteria, ultimately including 1, 792 publications from the WOSCC database and 41 publications from the PubMed database. The specific screening process is shown in **Figure 1**.

### 2.5 Bibliometric analysis software

Microsoft Excel 2019 was used for data cleaning and descriptive statistics. Bibliometrix was used for publication trends and bibliometric indicators; CiteSpace (v7.0) was used for collaboration networks, co-citation analysis, keyword co-occurrence, clustering, timeline visualization, and burst detection.

The parameters of CiteSpace (v7.0) software were set as follows: Time Span: 2016–2025; Slice Length: 1 year; Selection Criteria: g-index (k = 25); Pruning: Pathfinder, pruning sliced networks. After configuration, visualization and analysis were performed using different analytical elements as network nodes.

## 3 Results

### 3.1 Publication volume trends

**Figure 2:**
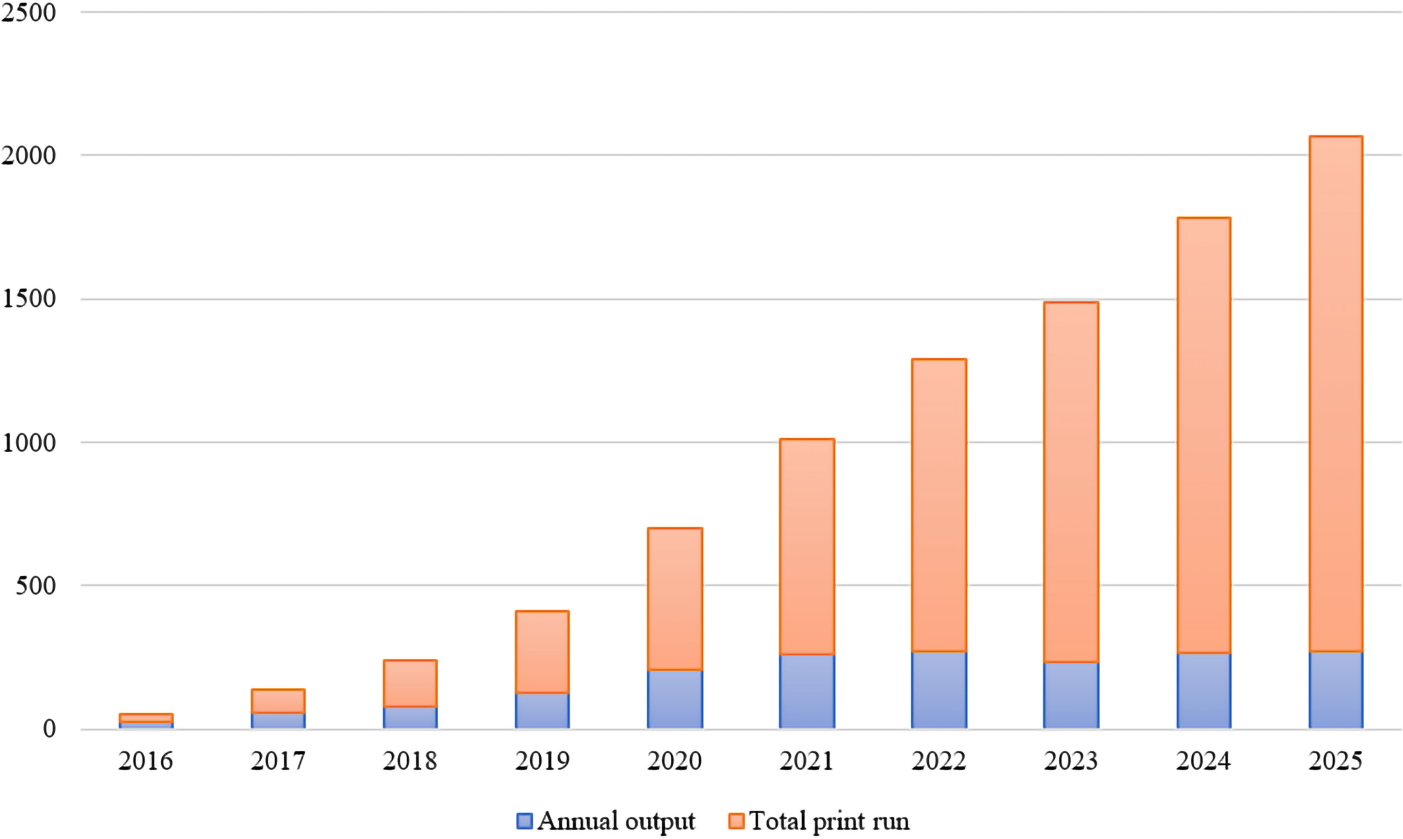
Annual trends in publications.

From 2016 to 2025, 1, 792 WoSCC publications were included. **Figure 2** illustrates publication trends. Annual output increased from 26 publications in 2016 to 273 publications in 2025. The number of publications increased gradually from 2016 to 2019, rose more rapidly between 2020 and 2022, and remained above 200 publications per year from 2023 to 2025. The cumulative number of publications exceeded 1, 000 by 2022 and reached 1, 792 by 2025. These results indicate sustained growth in research activity on ICI-related irAEs in lung cancer.

### 3.2 Collaboration and distribution by country/region

**Figure 3:**
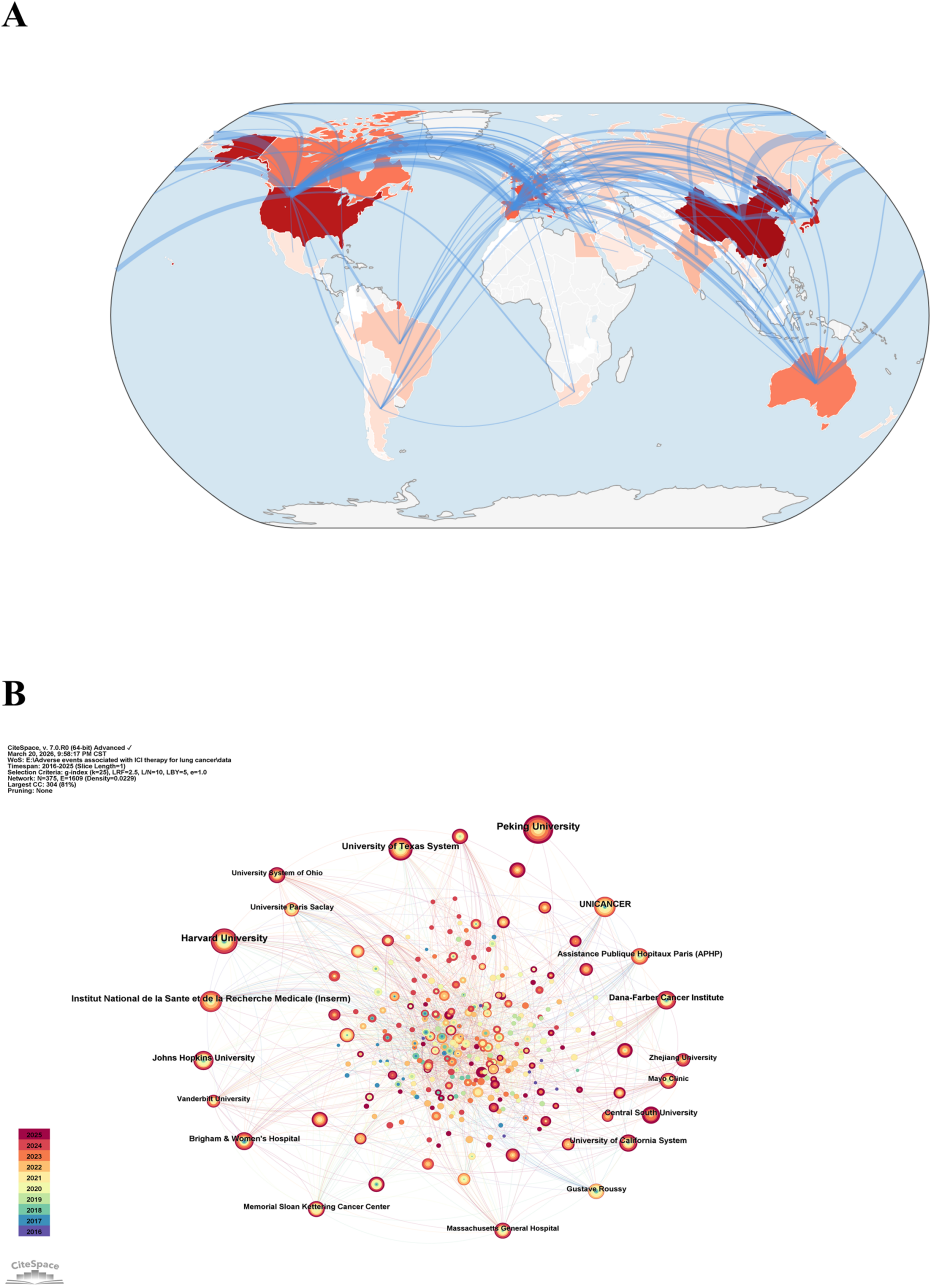
**(A)** View of cooperation relationships between countries/regions. **(B)** View of institutional collaboration relationships.

A total of 323 countries/regions contributed to this field. China had the highest number of publications (n = 626), followed by the United States (n = 419) and Japan (n = 315). **Figure 3A** illustrates a network analysis view of countries/regions; **Table 1** shows the top 15 countries/regions by publication volume. In the collaboration network, the United States showed the highest betweenness betweenness centrality (0.42), followed by Spain and Germany (both 0.11). China had a betweenness centrality value of 0.09, indicating that its network betweenness centrality was lower than its publication output. The collaboration map showed frequent links among the United States, major European countries, and selected Asia-Pacific countries, whereas countries in South America and Africa contributed fewer publications and had fewer collaboration links.

**Table 1:** Top 15 countries/regions by number of publications.

Rank	Count	Betweenness centrality	Country/regions
1	626	0.09	CHINA
2	419	0.42	USA
3	315	0.01	JAPAN
4	119	0.06	ITALY
5	103	0.08	FRANCE
6	65	0.11	SPAIN
7	55	0.11	GERMANY
8	50	0.07	ENGLAND
9	50	0.02	CANADA
10	44	0.07	AUSTRALIA
11	41	0.02	SOUTH KOREA
12	37	0.05	SWITZERLAND
13	30	0.01	GREECE
14	27	0.03	NETHERLANDS
15	25	0.01	BELGIUM

In terms of publication volume, China ranked first globally with 626 publications, accounting for 34.93% of the total included literature, making it the world’s largest producer of research in this field; the United States ranked second globally with 419 publications, accounting for 23.38%. Together, China and the United States accounted for over 58% of the total publications, together accounting for more than half of the included publications. Japan ranked third globally with 315 publications, making it the second-largest research producer in East Asia after China, with a publication output significantly higher than that of subsequent countries. Following this is a cluster of European nations, with Italy and France ranking fourth and fifth with 119 and 103 publications, respectively, serving as the core research producers in this field within Europe; European countries such as Spain, Germany, the United Kingdom, and Switzerland, as well as Asia-Pacific nations like Australia and South Korea, all published fewer than 100 papers, ranking in the lower half of the top 15. Overall, research output in this field is primarily concentrated in East Asia, North America, and Western Europe, while countries in regions such as South America and Africa have minimal research output and very low participation in this field.

The United States’ betweenness betweenness centrality was 0.42, far surpassing other countries, indicating that it occupied a prominent bridging position in the international collaboration network. Spain and Germany both have a Betweenness centrality of 0.11, tying for second place globally, and serve as important hubs for academic collaboration within the European region. China’s Betweenness centrality is 0.09, ranking fourth globally. Although it possesses a certain capacity to influence international collaboration, its network betweenness centrality was lower than expected relative to its publication output. France, the United Kingdom, and Australia have centralities of 0.08, 0.07, and 0.07, respectively, and exert a certain degree of influence in both regional and cross-regional cooperation. It is worth noting that although Japan ranks third globally with 315 publications, its betweenness centrality is only 0.01, placing it at the bottom among the top 15 countries by publication volume. This suggests that while Japan has a high volume of research output, its participation in international collaboration is extremely low, and it occupied a relatively peripheral position in the international collaboration network despite its high publication output.

The international collaboration network map reveals that the global collaboration network in this field exhibits a typical “core-periphery” distribution. The blue lines in the figure represent collaborative relationships between countries; the denser the lines, the higher the frequency of collaboration. Notably, the United States maintains dense collaborative links with major research nations across all continents, with its network spanning North America, Europe, the Asia-Pacific, South America, and Oceania. This pattern was consistent with the relatively high betweenness centrality value of the United States. China’s collaborative links primarily cover the Asia-Pacific region (Japan, South Korea, Australia), North America (the United States), and major research nations in Western Europe, indicating a foundation for cross-regional cooperation. However, the density of its collaborative links, its reach, and its role as a hub fall short of those of the United States. Western European countries have formed a highly dense intra-regional collaboration network, while maintaining frequent transatlantic collaboration with the United States, forming the world’s second-largest collaboration cluster. Countries such as Spain, Germany, France, and Italy play a central connecting role in European regional collaboration. In contrast, most countries in South America and Africa have minimal research output in this field, with only a few engaging in sporadic collaboration with global core nodes.

### 3.3 Distribution and collaboration among institutions

A total of 375 institutions were identified, with 1, 609 collaboration links and a network density of 0.0229. **Figure 3B** illustrates a network analysis view of institutions; **Table 2** shows the top 15 institutions by number of publications. The largest connected component contained 304 nodes. Peking University ranked first in publication output (n = 92), followed by Harvard University (n = 78), the University of Texas System (n = 60), Inserm (n = 56), and UNICANCER (n = 50). Gustave Roussy had the highest institutional betweenness betweenness centrality (0.08), followed by Inserm (0.06), Harvard University (0.05), and Dana-Farber Cancer Institute (0.04). Overall, Chinese institutions contributed a substantial number of publications, whereas European and North American institutions showed relatively higher network betweenness centrality.

**Table 2:** Top 15 institutions by number of publications.

Rank	Count	Betweenness centrality	Institutions
1	92	0.01	Peking University
2	78	0.05	Harvard University
3	60	0.03	University of Texas System
4	56	0.06	Institut National de la Sante et de la Recherche Medicale (Inserm)
5	50	0.03	UNICANCER
6	46	0.04	Dana-Farber Cancer Institute
7	43	0	Johns Hopkins University
8	39	0.03	Brigham & Women’s Hospital
9	36	0.02	Assistance Publique Hopitaux Paris
10	34	0.02	Central South University
11	34	0.03	University of California System
12	33	0.03	Memorial Sloan Kettering Cancer Center
13	32	0	Shandong First Medical University & Shandong Academy of Medical Sciences
14	32	0	Sichuan University
15	31	0.08	Gustave Roussy

In terms of publication volume, research output in this field exhibits a significant concentration at the top: the top 15 institutions collectively published 701 papers, accounting for 39.12% of the total included literature. These institutions were the most productive contributors in the dataset and can be divided into three distinct tiers. First Tier (70 or more publications): Peking University ranks first globally with 92 publications, making it the most prolific research institution in this field; Harvard University ranks second globally with 78 publications, making it the most prolific institution in Europe and the Americas. These two institutions constitute the first tier of global research output in this field. Second Tier (50–69 publications): This tier includes the University of Texas System (60 publications), the Institut National de la Santé et de la Recherche Médicale (Inserm) (56 publications), and UNICANCER (50 publications). All are leading medical research institutions in Europe and the United States and represent the core backbone of research in this field. Third tier (30–49 publications): A total of 10 institutions, including 6 from Europe and the United States: Dana-Farber Cancer Institute (46 publications), Johns Hopkins University (43 publications), Brigham & Women’s Hospital (39 publications), University of California System (34 publications), Memorial Sloan Kettering Cancer Center (33 publications), and Gustave Roussy (31 publications); and 4 Chinese institutions, including Central South University (34 papers), Shandong First Medical University & Shandong Academy of Medical Sciences (32 papers), Sichuan University (32 papers), and Zhejiang University (31 papers), indicating that Chinese institutions have become a significant component of global research output in this field.

In terms of betweenness centrality, Gustave Roussy showed the highest betweenness betweenness centrality among institutions, with a betweenness centrality of 0.08, ranking first across all fields; it serves as the core hub connecting Europe with other global research clusters. The Institut National de la Santé et de la Recherche Médicale (Inserm) ranks second with a betweenness centrality of 0.06, acting as a core node within the European research cluster. The core hubs in North America are Harvard University (Betweenness centrality 0.05) and the Dana-Farber Cancer Institute (Betweenness centrality 0.04); the University of Texas System, UNICANCER, Brigham & Women’s Hospital, the University of California System, and Memorial Sloan Kettering Cancer Center all have a Betweenness centrality of 0.03. Together, these European and North American institutions occupied relatively central positions in the institutional collaboration network.

In terms of spatial clustering characteristics within the network, global research institutions in this field have formed three regional clusters with clearly defined boundaries and close internal collaboration, while collaborative links between clusters exhibit significant regional differentiation. Among these, the North American core cluster is centered around leading U.S. institutions such as Harvard University, the University of Texas System, the Dana-Farber Cancer Institute, and Johns Hopkins University. Connections between nodes within this cluster are highly dense, and the level of collaboration is the highest, suggesting relatively dense intra-cluster collaboration within North America. At the same time, it maintains extensive cross-regional collaborative ties with the European cluster. The European core cluster is centered around French institutions such as the Institut National de la Santé et de la Recherche Médicale (Inserm), Gustave Roussy, and UNICANCER, extending its influence to research institutions across multiple European countries. With close internal collaboration, it serves as the second-largest core cluster in the global collaboration network and maintains stable transatlantic collaborative ties with the North American cluster. The Asia-Pacific cluster, centered around Chinese institutions such as Peking University, Zhejiang University, Central South University, and Sichuan University, has formed a relatively independent regional cooperation cluster. While regional collaboration among institutions within the cluster is active, there are very few cross-regional cooperation links with the North American and European core clusters. It exhibits the characteristic of “close intra-regional cooperation but insufficient international collaboration” and is an emerging research cluster in this field that has risen since 2019.

From the perspective of the nodes’ temporal color spectrum, the development of institutions in this field exhibits a clear temporal stratification: From 2016 to 2018, during the research inception phase, the core active institutions were primarily European and American institutions such as Harvard University, Memorial Sloan Kettering Cancer Center, Johns. Hopkins University, with nodes predominantly colored in cool tones. These institutions served as the pioneers of early research in this field, laying the core evidence-based foundation for its development; Starting in 2019, as research in the field entered a period of rapid expansion, Chinese institutions such as Peking University, Central South University, and Zhejiang University rose rapidly to prominence. These nodes are predominantly colored in warm tones, with publication volumes continuing to climb, becoming a major source of incremental research output in the field.

Overall, the global research landscape in this field is characterized by “Western institutions dominating core hubs, while Chinese institutions are rapidly increasing their publication output.” North American and European institutions showed higher network connectivity and betweenness betweenness centrality than most Asia-Pacific institutions. While Chinese institutions have achieved a breakthrough in publication volume, their cross-regional collaboration links and network betweenness centrality remained relatively limited. In the future, they must strengthen cross-regional collaboration with top global institutions to elevate their hub status within the global research network.

### 3.4 Analysis of source journals

**Figure 4:**
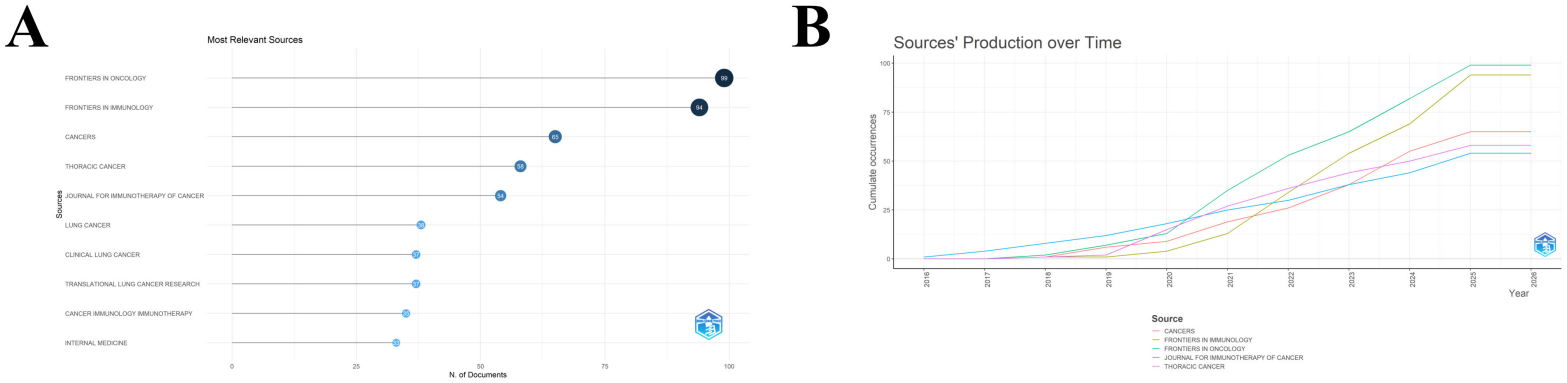
**(A)** Number of publications in leading journals. **(B)** Timeline of publications in leading journals.

A total of 414 journals contributed to the publication of works in this field. **Figure 4** illustrates the publication volume and cumulative trends of the major source journals.

In terms of publication distribution, the top 12 journals accounted for a total of 550 publications, representing 30.70% of the total included publications. Among them, FRONTIERS IN ONCOLOGY and FRONTIERS IN IMMUNOLOGY ranked first and second in terms of publication volume, publishing 99 and 94 relevant articles, respectively. Their publication volumes far exceeded those of other journals, making them the two journals with the highest number of included publications. The second-tier journals include Cancers (65 articles), Thoracic Cancer (58 articles), and Journal for Immunotherapy of Cancer (54 articles).among which Journal for Immunotherapy of Cancer is an authoritative specialized journal in the field of cancer immunotherapy, Cancers is an international core journal in general oncology, and Thoracic Cancer is a specialized journal in thoracic oncology. Together, these three form the core publishing platforms for general oncology, specialized oncology, and immunotherapy in this field. Additionally, specialized lung cancer journals such as Lung Cancer (38 articles), Clinical Lung Cancer(37 articles), and TRANSLATIONAL LUNG CANCER RESEARCH (37 articles), as well as specialized journals in cancer immunology such as CANCER IMMUNOLOGY IMMUNOTHERAPY (35 articles), also rank among the top 12 in terms of publication volume, further confirming the interdisciplinary nature of this field, which combines “specialized lung cancer diagnosis and treatment” with “basic and translational research in cancer immunology.”.

Looking at the temporal trends in the cumulative number of articles published in core journals, between 2016 and 2025, the cumulative output of the top five journals all showed a steady upward trend, closely aligning with the phased development characteristics of the field as a whole. This period can be divided into two distinct developmental stages. The period from 2016 to 2019 marked the field’s inception phase, during which the cumulative publication volumes of all five journals remained at relatively low levels, with a gradual growth trend. Overall research output in the field was limited during this stage, and the number of articles on relevant topics published in each journal was relatively small; Among them, the Journal for Immunotherapy of Cancer had a slightly higher cumulative publication volume than the other journals during this phase, indicating that this authoritative journal in the field of cancer immunotherapy had already focused on the topic of ICI-related adverse events during the early stages of research, indicating that it contributed relatively early publications to this topic. Starting in 2020, the cumulative publication volumes of all five journals entered a phase of rapid increase, was consistent with the rising research momentum in the field. Among them, FRONTIERS IN ONCOLOGY demonstrated the most significant growth rate, consistently leading all journals in cumulative publications after 2020, with a cumulative total approaching 100 articles by 2025;The growth trend of FRONTIERS IN IMMUNOLOGY closely aligns with that of the former. After 2020, its publication growth rate accelerated significantly, reaching a cumulative total of 94 articles by 2025 and firmly securing the second position. Meanwhile, Cancers, THORACIC CANCER, and JOURNAL FOR IMMUNOTHERAPY OF CANCER also exhibited a consistent, stable, and rapid growth trend in cumulative publication volume. Among these, Cancers’ growth rate accelerated further after 2022, while Thoracic Cancer and Journal for Immunotherapy of Cancer maintained a steady upward trajectory, with the cumulative number of articles for these three journals reaching 65, 58, and 54, respectively, by 2025.

### 3.5 Overlay map of journals

**Figure 5:**
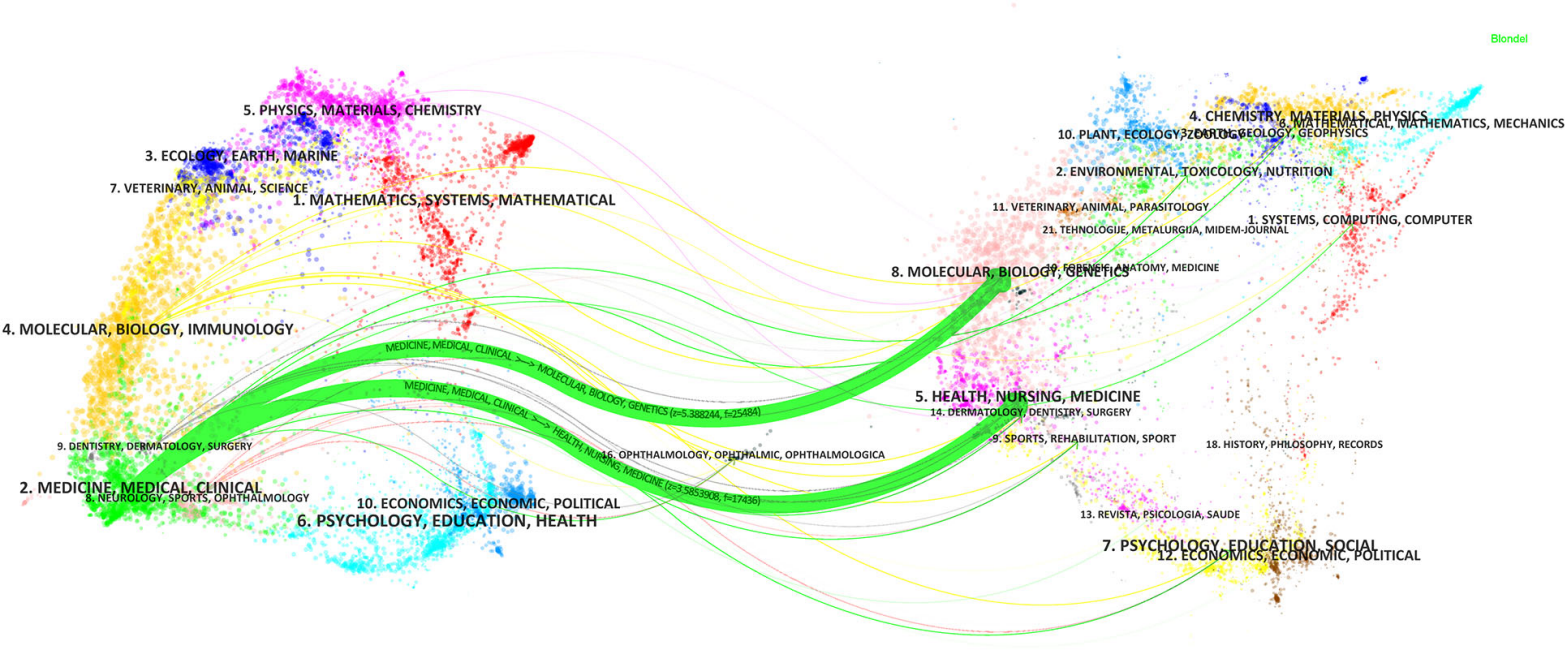
Overlay map of journals.

**Figure 5** presents the analysis results of the overlay map of journals. Using a standard map of 21 core disciplinary clusters worldwide as a base, the publications in this field exhibit an interdisciplinary distribution pattern: MEDICINE, MEDICAL, CLINICAL, and MOLECULAR, BIOLOGY, IMMUNOLOGY are the two core clusters with the most concentrated literature distribution. At the same time, there is extensive overlap with disciplines such as HEALTH, NURSING, MEDICINE; ENVIRONMENTAL, TOXICOLOGY, NUTRITION; and MOLECULAR, BIOLOGY, GENETICS, while some literature is distributed across interdisciplinary areas such as PSYCHOLOGY, EDUCATION, SOCIAL, SYSTEMS, COMPUTING, and COMPUTER.

The three most prominent thick green lines in the network clearly reveal the primary logic of knowledge evolution in this field: the main knowledge flow path is MEDICINE, MEDICAL, CLINICAL → MOLECULAR, BIOLOGY, GENETICS, reflecting the current situation where clinical problems drive the exploration of underlying mechanisms; The second most significant knowledge flow points toward HEALTH, NURSING, and MEDICINE, suggesting a connection between drug efficacy validation and comprehensive patient management; the third core flow points toward specialized fields such as OPHTHALMOLOGY, OPHTHALMIC, and OPHTHALMOLOGICA, which are associated with organ-specific immune-related adverse events.

### 3.6 Analysis of authors and author collaboration networks

**Figure 6:**
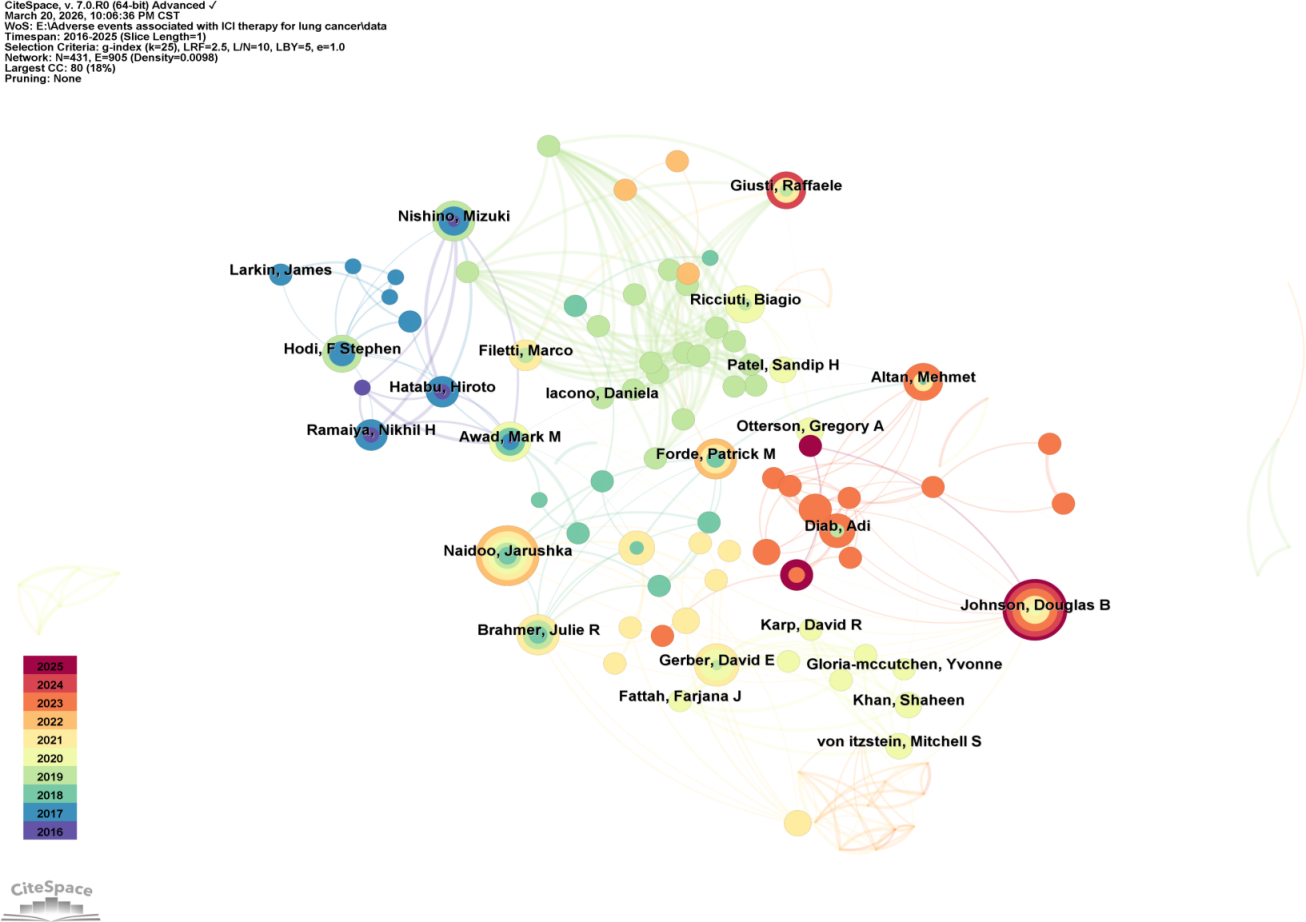
View of collaborations among authors.

The 1, 792 articles included in this study involved a total of 431 core authors who met the inclusion criteria. The author collaboration network is shown in **Figure 6**, and the publication counts and betweenness centrality statistics for the top 15 authors globally are presented in **Table 3**.

**Table 3:** Top 15 authors by number of publications.

Rank	Count	Betweenness centrality	Authors
1	17	0.02	Johnson, Douglas B
2	16	0.02	Zhang, Li
3	16	0.01	Naidoo, Jarushka
4	15	0.01	Xu, Yan
5	14	0	Wang, Mengzhao
6	11	0	Chen, Minjiang
7	9	0	Zhao, Jing
8	8	0	Zhou, Chengzhi
9	8	0	Zhong, Wei
10	8	0	Lambotte, Olivier
11	8	0	Michot, Jean-Marie
12	8	0.01	Gerber, David E
13	8	0	Liu, Ming
14	7	0	Forde, Patrick M
15	7	0	Nishino, Mizuki

The top 15 authors have published a cumulative total of 162 papers, accounting for 9.04% of the total included literature. Among them, Johnson, Douglas B ranked first globally with 17 publications; Zhang, Li and Naidoo, Jarushka both published 16 papers, tying for second place globally; Xu, Yan ranked fourth with 15 publications. These four authors had the highest publication counts among the included authors.

Based on node clustering characteristics, three core collaboration clusters can be identified: ① European Collaboration Cluster: Centered around Italian scholars such as Giusti, Raffaele; Iacono, Daniela; De Tursi, Michele; and Ricciuti, Biagio, this cluster features highly dense collaboration links among its nodes, showing relatively dense collaboration within this subgroup. ② North American Collaboration Cluster: Centered around Johnson, Douglas B, and extending to scholars such as Forde, Patrick M, Brahmer, Julie R, and Gerber, David E, this was one of the larger author collaboration subgroups observed in the network, maintaining collaborative ties with both the European and Asia-Pacific sub-clusters. The North American sub-cluster, centered on Hodi, F. Stephen, Ott, Patrick A., and Awad, Mark M., exhibits a node-time chromatogram primarily concentrated in the 2016–2019 period and represents the core group of contributors to the field’s early research.③ Asia-Pacific Collaboration Cluster: Centered on Japanese scholars such as Nishino, Mizuki and Hatabu, Hiroto, this cluster forms a relatively independent regional subnetwork with very few cross-regional collaborative links to the core clusters in Europe and the United States. None of the Chinese scholars ranked among the top 15 globally in terms of publication output appear in the global core interconnected subnetwork, and they have no significant collaborative ties with core research teams in Europe and the United States.

Regarding the evolution of the node chronological spectrum, during the initial research phase (2016–2018), the core active nodes were primarily scholars such as Hodi, F Stephen, Larkin, James, and Ramalya, Nikhil H; during the rapid growth phase (2019–2022), the core active nodes were primarily scholars such as Giusti, Raffaele, Ricciuti, Biagio, Johnson, Douglas B, and Naidoo, Jarushka; during the high-stability phase from 2023 to 2025, core active nodes were concentrated among scholars such as Johnson, Douglas B., Altan, Mehmet, and Diab, Adi. This indicates that these scholars remained consistently active throughout the entire research cycle, indicating sustained publication activity during the study period.

### 3.7 Co-citation analysis and identification of the core knowledge base

**Figure 7:**
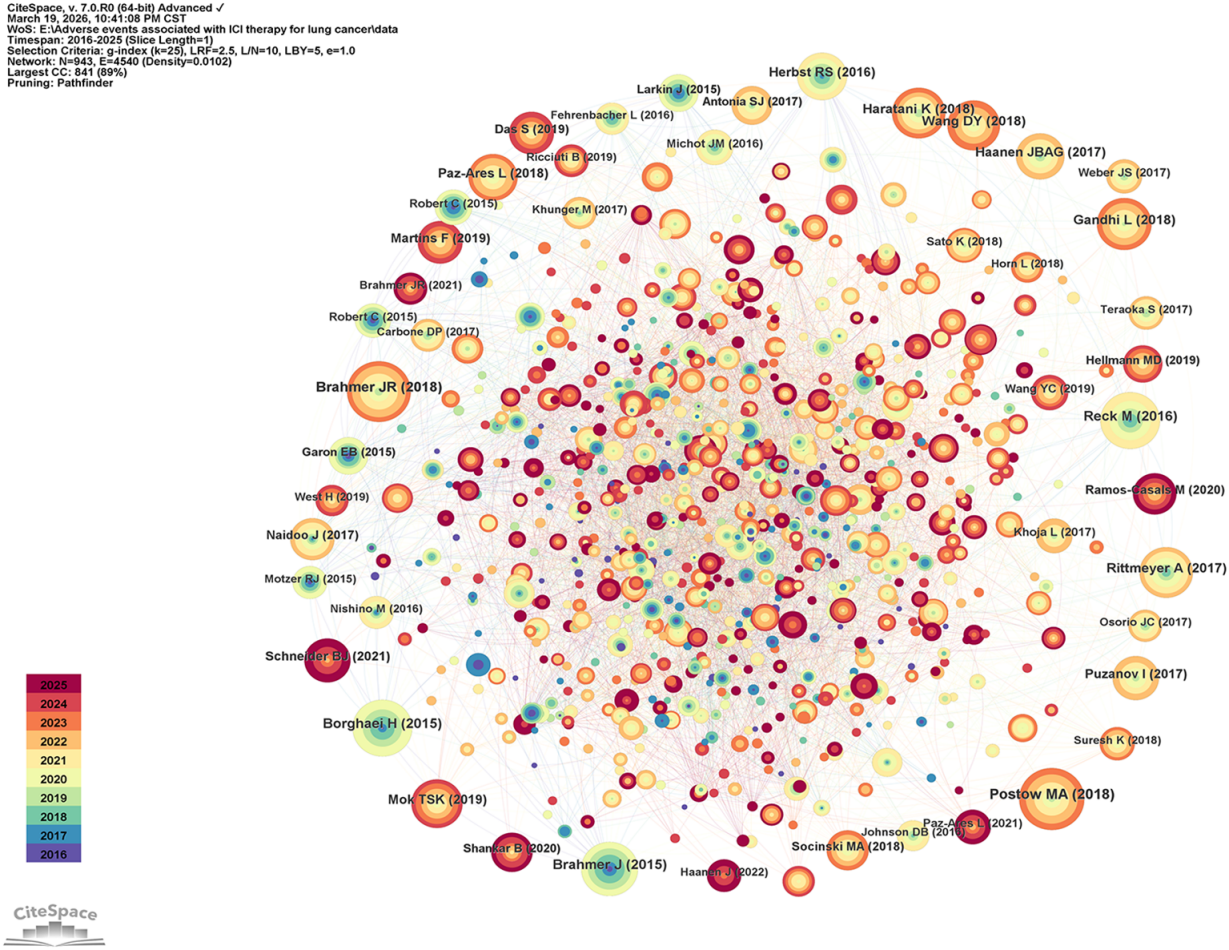
A network visualization of co-citation relationships among publications.

In this study, a co-citation network of the literature in this field was constructed using CiteSpace (v7.0). The results are shown in **Figure 7**, and the statistical results for the top 15 most frequently co-cited papers are presented in **Table 4**.

**Table 4:** Top 15 most frequently cited references.

Rank	Count	Betweenness centrality	References
1	255	0	Postow MA, 2018, NEW ENGL J MED, V378, P158, DOI 10.1056/NEJMra1703481
2	240	0	Brahmer JR, 2018, J CLIN ONCOL, V36, P1714, DOI 10.1200/JCO.2017.77.6385
3	212	0.05	Borghaei H, 2015, NEW ENGL J MED, V373, P1627, DOI 10.1056/NEJMoa1507643
4	208	0	Reck M, 2016, NEW ENGL J MED, V375, P1823, DOI 10.1056/NEJMoa1606774
5	190	0.03	Brahmer J, 2015, NEW ENGL J MED, V373, P123, DOI 10.1056/NEJMoa1504627
6	180	0.01	Gandhi L, 2018, NEW ENGL J MED, V378, P2078, DOI 10.1056/NEJMoa1801005
7	174	0	Rittmeyer A, 2017, LANCET, V389, P255, DOI 10.1016/S0140-6736(16)32517-X
8	171	0	Haratani K, 2018, JAMA ONCOL, V4, P374, DOI 10.1001/jamaoncol.2017.2925
9	168	0.05	Wang DY, 2018, JAMA ONCOL, V4, P1721, DOI 10.1001/jamaoncol.2018.3923
10	158	0	Mok TSK, 2019, LANCET, V393, P1819, DOI 10.1016/S0140-6736(18)32409-7
11	152	0.12	Herbst RS, 2016, LANCET, V387, P1540, DOI 10.1016/S0140-6736(15)01281-7
12	142	0	Paz-Ares L, 2018, NEW ENGL J MED, V379, P2040, DOI 10.1056/NEJMoa1810865
13	141	0.01	Haanen JBAG, 2017, ANN ONCOL, V28, P119, DOI 10.1093/annonc/mdx225
14	132	0.01	Puzanov I, 2017, J IMMUNOTHER CANCER, V5, P0, DOI 10.1186/s40425-017-0300-z
15	129	0	Schneider BJ, 2021, J CLIN ONCOL, V39, P4073, DOI 10.1200/JCO.21.01440

The co-citation network contained 943 cited references and 4, 540 links, with a network density of 0.0102. The top co-cited references could be grouped into two main categories. The first category included registration trials of ICIs in lung cancer, such as CheckMate 057, KEYNOTE-024, KEYNOTE-189, and PACIFIC. The second category included guidelines, consensus statements, and reviews on irAE management, including the review by Postow et al. and the ASCO guideline by Brahmer et al. These references were frequently co-cited and were closely related to the clinical application of ICIs and the terminology, grading, and management of irAEs.

In terms of co-citation frequency, the top 15 publications each have a total citation count exceeding 129, indicating that these publications were frequently co-cited within the dataset. These references could be grouped into two major categories. The first category included landmark registration trials of ICIs in lung cancer on ICI therapy for lung cancer; these publications were all published into international medical journals such as The New England Journal of Medicine and The Lancet. They serve as the core evidence supporting the approval of ICIs indications across all treatment lines for lung cancer. Additionally, these studies were the first to systematically report the incidence, organ-specific patterns, and clinical characteristics of irAEs associated with different ICIs treatment regimens, providing baseline data and a reference framework for subsequent irAE-related research. Among these, the CheckMate 057 study published by Borghaei H et al. in 2015 (cited 212 times), the KEYNOTE-024 study published by Reck M et al. in 2016 (cited 208 times), the KEYNOTE-189 study published by Gandhi L et al. in 2018 (cited 180 times), and the PACIFIC study published by Mok TSK et al. in 2019 (cited 158 times), respectively demonstrated survival benefits of PD-1/PD-L1 inhibitors as monotherapy, in combination with chemotherapy, and as consolidation therapy following concurrent chemoradiotherapy in non-small cell lung cancer. These studies represent pivotal milestones in the widespread clinical adoption of ICIs for lung cancer and simultaneously provide large-sample registry data for the clinical characterization of irAEs (3, 4, 14, 15). The second category included guidelines, consensus statements, and reviews on irAE management. These publications serve as the main structure for the assessment, grading, and intervention of irAEs in clinical practice and research design, with consistently ranking among the top in the field in terms of citation frequency. Among them, the systematic review on irAEs by Postow MA et al., published in the New England Journal of Medicine in 2018, ranks first in the entire field with a total of 255 citations; The irAEs management guidelines by Brahmer JR et al., published in the Journal of Clinical Oncology in 2018, have a total citation count of 240, ranking second; these two publications systematically review the pathogenesis, clinical phenotypes, and management-related topics strategies for irAEs. They were among the most frequently co-cited references and are closely related to standardized terminology, grading, and management approaches for irAEs (6, 10).

In terms of betweenness betweenness centrality, KEYNOTE-010 had the highest value (0.12), suggesting a bridging position within the co-citation network (16). Borghaei H 2015 and Wang DY 2018 both have an betweenness centrality of 0.05, ranking jointly second in the entire field (7). Additionally, Brahmer J 2015 (Betweenness centrality 0.03) represents an early Phase I clinical trial of ICIs for non-small cell lung cancer. It was the first to clearly define the dosing regimen, safety profile, and dose-limiting toxicities of PD-1 inhibitors, providing a basis for the design of subsequent registration studies (17).

From the perspective of node-time chromatograms and spatial distribution, the core knowledge base in this field exhibits clear temporal stratification. Literature published between 2015 and 2018 occupies the core region of the co-citation network, characterized by large node volumes, highly dense connections, and predominantly cool color tones. This region serves as the central hub of the entire knowledge network, constituting the field’s most fundamental knowledge base. Publications from 2019 to 2021 are located in the middle layer of the network. These nodes are of moderate size and predominantly colored in yellow and orange tones. They represent the core literature of the field’s expansion phase, primarily focusing on the application of ICIs in the perioperative period and neoadjuvant therapy, as well as updates to irAEs management guidelines and real-world studies, thereby further expanding the boundaries of research in this field. Literature published between 2022 and 2025 is located in the outer region of the network, with relatively small nodes and predominantly warm colors.

### 3.8 Co-occurrence of keywords and cluster analysis of research hotspots

**Figure 8:**
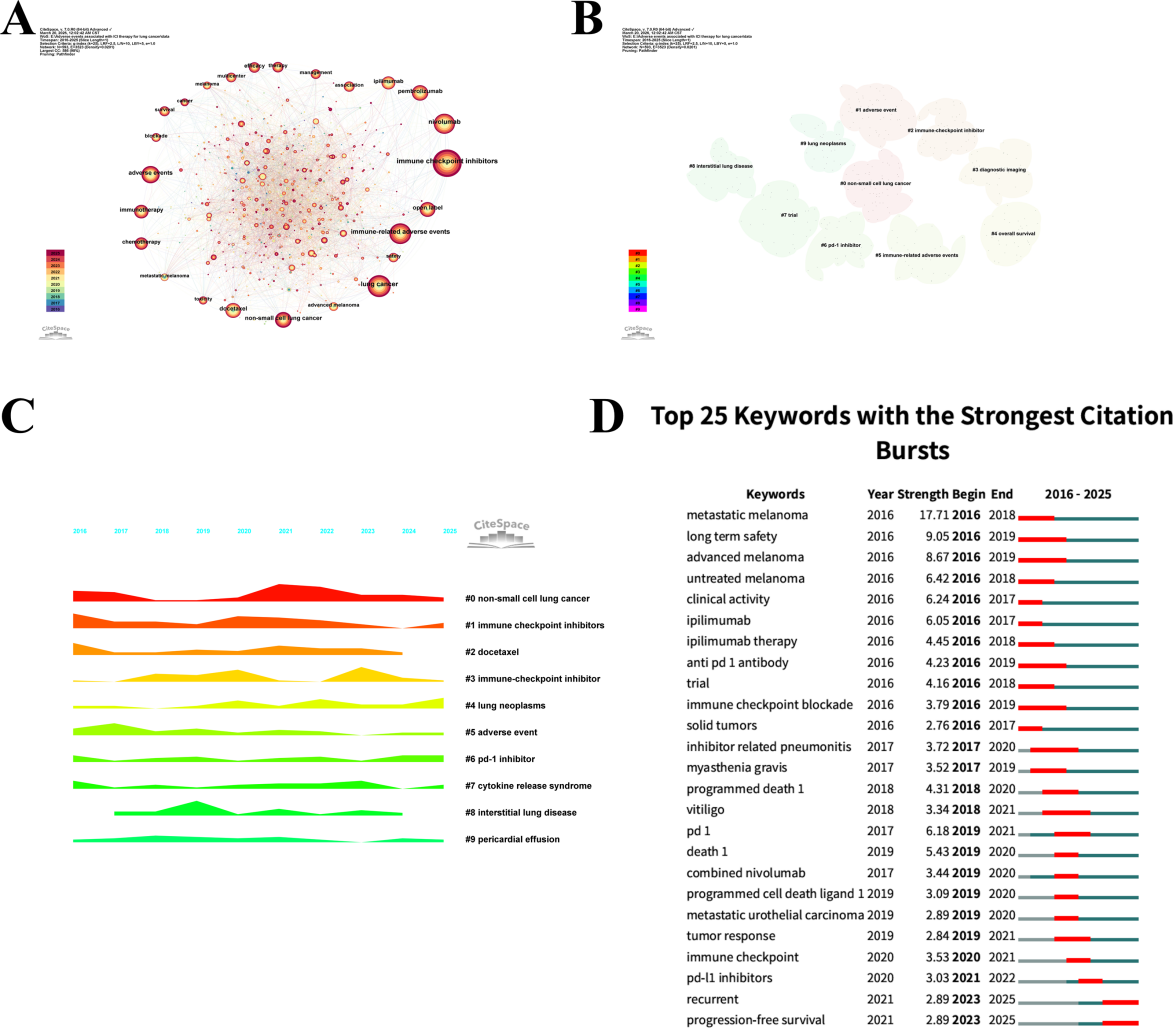
**(A)** Keyword co-occurrence network; **(B)** keyword clustering network; **(C)** keyword clustering landscape; **(D)** keyword burst analysis.

In this study, a keyword co-occurrence network was constructed using CiteSpace (v7.0) software, and topic clustering was performed using the Log-Likelihood Ratio (LLR) algorithm to systematically analyze the core research topics, the distribution of hotspots, and the evolution patterns of topics in this field. The results are shown in **Figures 8A, B**, and the top 15 keywords ranked by frequency and betweenness centrality are listed in **Table 5**.

**Table 5:** Top 15 keywords by frequency.

Rank	Count	Betweenness centrality	Keywords
1	547	0	nivolumab
2	546	0	immune checkpoint inhibitors
3	401	0	immune-related adverse events
4	395	0.01	adverse events
5	351	0.02	cell lung cancer
6	333	0.03	pembrolizumab
7	307	0.04	open label
8	305	0	docetaxel
9	291	0.03	lung cancer
10	268	0.01	immune checkpoint inhibitor
11	264	0	immunotherapy
12	243	0.01	ipilimumab
13	239	0.01	non-small cell lung cancer
14	213	0.02	chemotherapy
15	178	0.06	efficacy

The results show that the keyword co-occurrence network comprises 593 nodes and 3, 523 edges, with a network density of 0.0201. The largest connected subgraph contains 586 nodes, accounting for 98% of the total nodes. This suggests that research themes in this field are highly focused and that the knowledge system is tightly integrated, with no obvious divisions between different research directions, indicating that most keywords were connected within a single network. In the network diagram, node size is positively correlated with keyword frequency, and node color corresponds to the year of the keyword’s first appearance. Cooler tones (2016–2018) represent early-stage core themes in the field, while warmer tones (2019–2025) represent emerging and deeply explored hot topics; the denser the connections between nodes, the higher the degree of association among research themes.

The keyword co-occurrence network contained 593 nodes and 3, 523 edges, with a network density of 0.0201. The most frequent keywords were “nivolumab,” “immune checkpoint inhibitors,” “immune-related adverse events, ” “adverse events, ” “cell lung cancer,” and “pembrolizumab.” These keywords reflected three broad topic areas: ICI agents and treatment strategies, lung cancer populations, and safety-related outcomes. Keywords related to irAEs and adverse events appeared with high frequency, indicating sustained attention to safety-related topics in the included literature.

In terms of betweenness centrality, the betweenness centrality scores of the top 15 keywords are highly consistent with the thematic logic of the clustering map: Efficacy (Betweenness centrality 0.06): The term “efficacy” had the highest betweenness centrality among the top keywords, indicating a bridging role between intervention-, population-, and safety-related terms, corresponding to Cluster #4 (overall survival). It connects three themes: intervention drugs, patient populations, and safety studies. This suggests that all irAE-related research is premised on ensuring antitumor efficacy for patients, with the ultimate goal of achieving a balance between therapeutic benefits and risks—a core logical thread running through the entire field. Open Label (Betweenness centrality 0.04): Corresponding to Cluster #7 (Trial), it connected several terms related to study design and clinical evaluation. This indicates that open-label, multicenter RCTs are the primary source of core evidence-based data in this field. Whether verifying the efficacy of ICIs or analyzing the incidence and risk factors of irAEs, high-quality clinical trials provide the core data support. Pembrolizumab (Betweenness centrality 0.03) and lung cancer (Betweenness centrality 0.03): As representatives of the core intervention drug and disease population, respectively, they serve as central nodes connecting drug development, clinical research, and adverse event management. Corresponding to clusters #6 and #0, they form the main structure of the research network. It is worth noting that although “adverse events” (Betweenness centrality 0.01) and “immune-related adverse events” did not rank among the top 5 in Betweenness centrality, they co-occur with over 90% of the nodes in the network, making them one of the most widely covered research topics in the field. This suggests that irAE-related research has permeated the entire process of ICIs treatment for lung cancer, rather than being an independent branch of research.

LLR clustering identified 10 valid clusters. The largest clusters were related to non-small cell lung cancer, adverse events, immune checkpoint inhibitors, diagnostic imaging, overall survival, immune-related adverse events, PD-1 inhibitors, trials, interstitial lung disease, and lung neoplasms. These clusters indicate that the keyword structure was mainly organized around disease type, ICI treatment, safety outcomes, efficacy endpoints, and imaging-related evaluation.

From the temporal chromatographic characteristics of keywords, research hotspots in this field exhibit a clear evolutionary pattern. During the initial research phase from 2016 to 2018, frequently observed topic were concentrated in the foundational framework module, centered on validating the efficacy of ICIs in treating lung cancer, corresponding to the cool-toned “ “ nodes clustered in the core region of the network; Starting in 2019, with the widespread clinical application of ICIs in lung cancer, research hotspots rapidly shifted toward the frequently observed topic module. The volume of nodes related to irAEs continued to increase, and the density of connections significantly rose, with corresponding warm-colored nodes gradually extending toward the core region of the network—a pattern that was consistent with the increase in publication volume in this field. This evolutionary pattern suggests that, as ICIs become more widespread in lung cancer treatment, the focus of clinical research has shifted from “whether they are effective” to “how to safely and effectively manage the entire treatment process.” These findings suggest sustained attention to irAE-related management topics in recent publications.

### 3.9 Analysis of the evolution of research hotspots and burst trends

Based on keyword cluster timeline visualization and keyword burst analysis, this study systematically analyzes the dynamic evolution patterns, migration pathways, and cutting-edge trends of research hotspots in this field from 2016 to 2025. The results are shown in **Figures 8C, D**.

The keyword clustering landscape map clearly illustrates the changes in popularity and development cycles of 10 core research themes between 2016 and 2025. The width of the bands in the figure is positively correlated with the research popularity in the corresponding years, while the continuity of the bands represents the persistence of research on each theme. The results show that the evolution of research themes in this field can be divided into three major clusters: ① Basic framework theme cluster: covering #0 non-small cell lung cancer, #1 immune checkpoint inhibitors, #2 docetaxel, #4 lung neoplasms, and #6 PD-1 inhibitor. These themes emerged as early as the initial research phase in 2016 and constitute the core underlying framework spanning the entire research cycle, with research interest consistently remaining at a high level. Among them, #0 non-small cell lung cancer, as the core research population in the field, reached its peak in 2020–2021 and has remained stable since then.② Core cluster of adverse event themes: #5 adverse event. This theme began in 2016, with research interest rising steadily and continuously without interruption. It entered a plateau phase at a high level after 2020 and has maintained stable interest through 2025, fully aligning with the overall growth trend in the field’s publication volume. This suggests that adverse events and safety management are core research hotspots throughout the field’s entire development cycle and remain a key area of ongoing concern in clinical practice.③ Sub-theme clusters of specific immune-related adverse events: These include #7 cytokine release syndrome, #8 interstitial lung disease, and #9 pericardial effusion. These topics focus on organ-specific, high-risk irAEs associated with ICI therapy, which gradually entered the research spotlight between 2016 and 2017. Among them, research interest in #8 interstitial lung disease peaked in 2018, making it the most closely watched fatal irAE during this phase; since then, research interest has remained stable. Research interest in rare, high-risk cardiovascular irAEs such as #7 cytokine release syndrome and #9 pericardial effusion remained consistent throughout the period, with a notable increase in activity observed after 2023.These findings suggest that, as clinical experience accumulates, research in this field has shifted from broad statistical analyses of overall adverse events toward the precise identification, risk prediction, and clinical management of organ-specific, rare, and highly lethal irAEs, with the depth of research on these specialized topics continuing to increase.

Keyword burst analysis showed that early burst terms were mainly related to melanoma, ipilimumab, and immune checkpoint blockade. During the subsequent period, burst terms related to lung cancer-specific irAEs, including inhibitor-related pneumonitis and myasthenia gravis, appeared. More recent burst terms included “recurrent” and “progression-free survival, ” suggesting increased recent attention to recurrent disease and survival-related outcomes in the dataset. These burst terms should be interpreted as indicators of temporal research attention rather than direct evidence of future clinical directions.

In summary, from 2016 to 2025, research trends in the field of adverse events associated with ICI therapy for lung cancer have followed a clear four-stage progression: “originating in melanoma—focusing on lung cancer-specific irAEs—extending to the relationship between efficacy and safety—and expanding to the long-term prognosis of patients with recurrent disease. “Comprehensive management of adverse events remains a core focus throughout the entire research lifecycle. Precision diagnosis and treatment of organ-specific, high-risk irAEs represent a subfield requiring continued in-depth exploration, while safety assessments of immunotherapy for recurrent and advanced lung cancer, as well as analyses of the association between irAEs and long-term patient survival, were among the most recent burst-related topics in this dataset.

### 3.10 Descriptive mapping of PubMed clinical-study records

To complement the WoSCC-based bibliometric analysis, 41 PubMed records classified as clinical studies were analyzed descriptively. This supplementary analysis focused on the distribution of clinical study types, disease settings, intervention regimens, irAE categories, and keyword-based topic groups. It was not intended to evaluate clinical efficacy, compare treatment regimens, or synthesize safety evidence.

**Figure 9:**
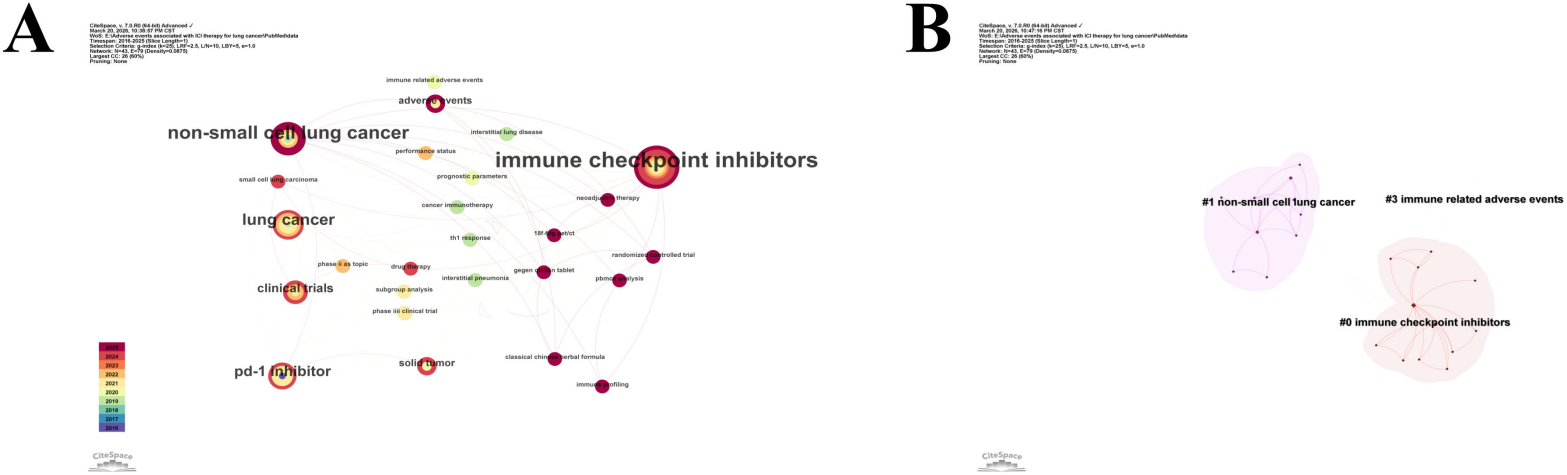
**(A)** Co-occurrence analysis network of clinical trial keywords; **(B)** Keywords clustering network of clinical trial.

Keywords serve as a crucial means of reflecting research hotspots and cutting-edge trends in the field. We explored the hotspots and advancements in clinical research by constructing keyword co-occurrence and clustering analysis visualizations, as well as analyzing high-frequency keywords. The results are shown in **Figure 9**. The basic information of the 41 clinical studies included is presented in **Supplementary Material 2**.

The PubMed keyword network showed several descriptive topic groups. First, terms related to the main intervention and disease setting, including “immune checkpoint inhibitors, ” “PD-1 inhibitor, ” “non-small cell lung cancer, ” “lung cancer, ” and “small cell lung carcinoma, ” formed the main keyword structure. Second, terms related to study design and clinical evaluation, including “clinical trials, ” “phase II, ” “phase III clinical trial, ” “randomized controlled trial, ” and “subgroup analysis, ” indicated that the included PubMed records covered different clinical-study designs. Third, safety-related terms, including “adverse events, ” “immune-related adverse events, ” “interstitial lung disease, ” and “interstitial pneumonia, ” appeared as important nodes in the network. Additional topic terms, such as “neoadjuvant therapy, ” “18F-FDG PET/CT, ” “PBMC analysis, ” and “classical Chinese herbal formulas, ” were observed in a smaller number of records. These terms were interpreted only as descriptive topic signals within the PubMed dataset.

In terms of temporal distribution, the PubMed keyword map suggested that earlier records were mainly associated with PD-1/PD-L1 inhibitors, NSCLC, and clinical trials. Records published after 2019 more frequently involved terms related to irAEs, interstitial pneumonia, subgroup analysis, and perioperative or neoadjuvant treatment settings. In the most recent records, several terms related to immune profiling, PBMC analysis, imaging-based evaluation, and herbal formula-related interventions appeared. Because this supplementary analysis was based on keyword co-occurrence and descriptive classification only, these findings should be interpreted as topic distributions within the PubMed clinical-study subset rather than evidence of clinical effectiveness, safety superiority, or practice-changing directions.

## 4 Discussion

### 4.1 Overview of research results

Based on 1, 792 publications from the WoSCC database published between 2016 and 2025, supplemented by clinical research data from the PubMed database, this study employed bibliometric and visualization analysis methods to systematically map the global research landscape in the field of ICI-related adverse events for lung cancer.

The findings show sustained growth in publication output, geographically uneven collaboration patterns, frequently co-cited references related to ICI trials and irAE management, and keyword clusters centered on lung cancer, ICI therapy, adverse events, clinical trials, and interstitial lung disease.

### 4.2 Global research landscape and collaboration models

The results of this study show that over the past decade, the annual publication volume in this field has grown from 26 articles in 2016 to 273 articles in 2025, representing a cumulative increase of 6, 792.31%, with rapid increase beginning after 2020.This trend aligns closely with the clinical adoption of ICIs in lung cancer treatment: Between 2019 and 2020, multiple ICIs—including pembrolizumab, nivolumab, and durvalumab—were successively approved for first-line, neoadjuvant, and adjuvant treatment of lung cancer and incorporated into standard treatment regimens by authoritative domestic and international guidelines such as the National Comprehensive Cancer Network and Chinese Society of Clinical Oncology (18–20).The widespread adoption of ICIs across the entire course of lung cancer treatment has made the standardized management of irAEs a core clinical necessity, directly driving the rapid growth of related research.

The collaboration analysis showed that China contributed the largest number of publications, whereas the United States had the highest betweenness betweenness centrality. This difference suggests that publication output and network connectivity are not equivalent bibliometric dimensions. Similar patterns were observed at the institutional level: several Chinese institutions had high output, whereas European and North American institutions showed relatively higher network betweenness centrality. These findings indicate that future bibliometric studies may further examine how cross-regional collaboration affects knowledge dissemination in this topic.

Results at the institutional level further corroborate this pattern: although domestic institutions such as Peking University and Central South University rank among the top 15 globally in terms of publication volume, only Peking University and Central South University possess extremely low betweenness centrality; in contrast, European and American institutions such as Harvard University and the Gustave Roussy Cancer Center are not only high-output research entities but also core hubs in global collaboration networks.

### 4.3 Evolution of the disciplinary knowledge base and research hotspots

The co-citation analysis of the literature in this study reveals that the core knowledge base of this field consists of two pillars: first, landmark randomized controlled trials (RCTs) on ICIs for lung cancer treatment; and second, authoritative guidelines and systematic reviews on irAEs management. The two most frequently cited publications in the field are the systematic review on irAEs by Postow et al. published in the New England Journal of Medicine in 2018, and the ASCO clinical practice guidelines for irAEs management by Brahmer et al. published in the Journal of Clinical Oncology in 2018. These two publications were frequently co-cited and are closely related to standardized irAE terminology and management (6, 10). Meanwhile, landmark RCTs such as KEYNOTE-024, KEYNOTE-189, and PACIFIC not only established the standard of care for ICIs in lung cancer treatment but also provided large-sample baseline data on irAEs incidence, providing commonly cited safety data for subsequent studies (21–23).

The results of keyword co-occurrence and burst analysis clearly illustrate the four-phase migration pattern of research hotspots in this field: ① Initial Phase (2016–2018): Research originated from safety explorations of ICIs in the melanoma field; ② Rapid Expansion Phase (2017–2020): Research focus shifted to lung cancer-specific high-risk irAEs (particularly immune-related pneumonia);③ In-depth Development Phase (2019–2022): Research expanded to include association analyses between irAEs and antitumor efficacy;④ Recent burst-related topic Phase (2023–2025): Research focuses on safety assessments and long-term patient prognosis in recurrent or progressive lung cancer. This pattern appears to correspond to the broader clinical expansion of ICIs in lung cancer.

In the PubMed-based supplementary mapping, a small number of recent clinical-study records contained terms related to traditional Chinese medicine or herbal formula-based interventions (24–27). Because this analysis did not include study-quality assessment or evidence synthesis, these records were interpreted only as examples of topic diversification within the PubMed subset. No conclusions regarding the efficacy or safety of these interventions were drawn.

### 4.4 Research implications and future directions

The findings of this study should be interpreted as descriptive bibliometric evidence reflecting the structure, evolution, and emerging priorities of the research field, rather than as direct clinical recommendations. From a research-planning perspective, the results highlight several areas requiring further attention. First, although China has become a major contributor in terms of publication volume, international collaborative links remain relatively limited compared with those of the United States and major European research centers. Strengthening cross-regional and multicenter collaboration may improve the integration and visibility of future research. Second, keyword evolution and burst analyses suggest increasing attention to safety issues in recurrent or advanced lung cancer, indicating the need for prospective studies focusing on ICI rechallenge, sequential therapy, and long-term safety outcomes. Third, biomarker-related terms and organ-specific irAE terms, including pneumonitis, myocarditis, and neurological irAEs, appeared repeatedly in keyword and burst analyses, suggesting that these topics may deserve continued bibliometric and clinical research attention. Finally, intervention-related topics observed in the PubMed subset should be interpreted cautiously because this study did not assess clinical effectiveness or safety.

## 5 Limitations

Several limitations should be acknowledged. First, this study included only English-language publications indexed in WoSCC and PubMed, which may have led to the underrepresentation of studies published in other languages or regional databases. Second, conference abstracts, guidelines, and grey literature were excluded. Because emerging safety signals and new irAE management strategies are often initially reported at major oncology conferences, this exclusion may underestimate the most recent developments in the field. Third, bibliometric indicators such as citation frequency, betweenness centrality, and keyword bursts reflect structural properties of the literature network and research attention, but they do not directly indicate study quality, clinical efficacy, or causal importance. Fourth, the PubMed-based analysis was designed only as supplementary clinical-topic mapping and should not be interpreted as a systematic review or quantitative synthesis of clinical evidence. Therefore, the findings should be interpreted as exploratory evidence for understanding research trends and planning future investigations.

## 6 Conclusions

This bibliometric study mapped the global research landscape, knowledge base, and thematic evolution of ICI-related irAEs in lung cancer from 2016 to 2025. The field has expanded rapidly, with research hotspots shifting from early pan-tumor safety exploration to lung cancer-specific high-risk irAEs, efficacy–safety associations, and long-term safety concerns in recurrent or advanced populations. The results also reveal an imbalance between publication output and collaborative network betweenness centrality, suggesting the need for stronger international and cross-institutional collaboration. The findings indicate several topics that may warrant further investigation, including safety-focused studies in recurrent, advanced, and underrepresented lung cancer populations, biomarker-related research, and organ-specific high-risk irAEs. Because the PubMed component was descriptive, intervention-related topics identified in that subset should not be interpreted as evidence-based clinical recommendations.

## Data Availability

The original contributions presented in the study are included in the article/**Supplementary Material**. Further inquiries can be directed to the corresponding author.
