# Supplementary material for: Current status and trends of immune-related adverse events in lung cancer treated with immune checkpoint inhibitors: a bibliometric analysis of the past decade (2016–2025)
Source: Front Immunol. 2026 Jun 2;17:1846212. doi: 10.3389/fimmu.2026.1846212 (PMC13269323; doi:10.3389/fimmu.2026.1846212)
Supplement: Supplementary file 1 [file Table1.docx]

Table 1: Search Strategy for the WOSCC Database

| **No** | **Search terms** | **Results** |
| --- | --- | --- |
| #1 | TS=("Immune Checkpoint Inhibitors" OR "Checkpoint Inhibitors, Immune" OR "Immune Checkpoint Blockers" OR "Checkpoint Blockers, Immune" OR "Immune Checkpoint Inhibitor" OR "Checkpoint Inhibitor, Immune" OR "CTLA-4 Inhibitors" OR "CTLA 4 Inhibitors" OR "Cytotoxic T-Lymphocyte-Associated Protein 4 Inhibitors" OR "Cytotoxic T Lymphocyte Associated Protein 4 Inhibitors" OR "Cytotoxic T-Lymphocyte-Associated Protein 4 Inhibitor" OR "Cytotoxic T Lymphocyte Associated Protein 4 Inhibitor" OR "CTLA-4 Inhibitor" OR "CTLA 4 Inhibitor" OR "PD-1 Inhibitors" OR "PD 1 Inhibitors" OR "Programmed Cell Death Protein 1 Inhibitor" OR "Programmed Cell Death Protein 1 Inhibitors" OR "PD-1 Inhibitor" OR "Inhibitor, PD-1" OR "PD 1 Inhibitor" OR "Immune Checkpoint Blockade" OR "Checkpoint Blockade, Immune" OR "Immune Checkpoint Inhibition" OR "Checkpoint Inhibition, Immune" OR "PD-L1 Inhibitors" OR "PD L1 Inhibitors" OR "Programmed Death-Ligand 1 Inhibitors" OR "Programmed Death Ligand 1 Inhibitors" OR "PD-L1 Inhibitor" OR "PD L1 Inhibitor" OR "PD-1-PD-L1 Blockade" OR "Blockade, PD-1-PD-L1" OR "PD 1 PD L1 Blockade" OR "ICI") AND Index Data=(2016-01-01 to 2025-12-31) | 60388 |
| #2 | TS=("Immune-Related Adverse Events" OR "Immune Related Adverse Events" OR "Immune-Related Adverse Event" OR "Immune Related Adverse Event" OR "Adverse Events, Immune-Related" OR "Adverse Event, Immune-Related" OR "irAEs" OR "Adverse event") AND Index Data=(2016-01-01 to 2025-12-31) | 35446 |
| #3 | TS=("Lung Neoplasms" OR "Pulmonary Neoplasms" OR "Neoplasms, Lung" OR "Lung Neoplasm" OR "Neoplasm, Lung" OR "Neoplasms, Pulmonary" OR "Neoplasm, Pulmonary" OR "Pulmonary Neoplasm" OR "Lung Cancer" OR "Cancer, Lung" OR "Cancers, Lung" OR "Lung Cancers" OR "Pulmonary Cancer" OR "Cancer, Pulmonary" OR "Cancers, Pulmonary" OR "Pulmonary Cancers" OR "Cancer of the Lung" OR "Cancer of Lung") AND Index Data=(2016-01-01 to 2025-12-31) | 205173 |
| #4 | #1 AND #2 AND #3 | 1849 |

Table 2: Search strategy for the PubMed database

| **No** | **Search terms** | **Results** |
| --- | --- | --- |
| #1 | "Immune Checkpoint Inhibitors"[MeSH] | 19330 |
| #2 | "Checkpoint Inhibitors, Immune"[Title/Abstract] OR "Immune Checkpoint Blockers"[Title/Abstract] OR "Checkpoint Blockers, Immune"[Title/Abstract] OR "Immune Checkpoint Inhibitor"[Title/Abstract] OR "Checkpoint Inhibitor, Immune"[Title/Abstract] OR "CTLA-4 Inhibitors"[Title/Abstract] OR "CTLA 4 Inhibitors"[Title/Abstract] OR "Cytotoxic T-Lymphocyte-Associated Protein 4 Inhibitors"[Title/Abstract] OR "Cytotoxic T Lymphocyte Associated Protein 4 Inhibitors"[Title/Abstract] OR "Cytotoxic T-Lymphocyte-Associated Protein 4 Inhibitor"[Title/Abstract] OR "Cytotoxic T Lymphocyte Associated Protein 4 Inhibitor"[Title/Abstract] OR "CTLA-4 Inhibitor"[Title/Abstract] OR "CTLA 4 Inhibitor"[Title/Abstract] OR "PD-1 Inhibitors"[Title/Abstract] OR "PD 1 Inhibitors"[Title/Abstract] OR "Programmed Cell Death Protein 1 Inhibitor"[Title/Abstract] OR "Programmed Cell Death Protein 1 Inhibitors"[Title/Abstract] OR "PD-1 Inhibitor"[Title/Abstract] OR "Inhibitor, PD-1"[Title/Abstract] OR "PD 1 Inhibitor"[Title/Abstract] OR "Immune Checkpoint Blockade"[Title/Abstract] OR "Checkpoint Blockade, Immune"[Title/Abstract] OR "Immune Checkpoint Inhibition"[Title/Abstract] OR "Checkpoint Inhibition, Immune"[Title/Abstract] OR "PD-L1 Inhibitors"[Title/Abstract] OR "PD L1 Inhibitors"[Title/Abstract] OR "Programmed Death-Ligand 1 Inhibitors"[Title/Abstract] OR "Programmed Death Ligand 1 Inhibitors"[Title/Abstract] OR "PD-L1 Inhibitor"[Title/Abstract] OR "PD L1 Inhibitor"[Title/Abstract] OR "PD-1-PD-L1 Blockade"[Title/Abstract] OR "Blockade, PD-1-PD-L1"[Title/Abstract] OR "PD 1 PD L1 Blockade"[Title/Abstract] OR "ICI"[Title/Abstract] | 48068 |
| #3 | #1 OR #2 | 54857 |
| #4 | "Immune-Related Adverse Events"[Title/Abstract] OR "Immune Related Adverse Events"[Title/Abstract] OR "Immune-Related Adverse Event"[Title/Abstract] OR "Immune Related Adverse Event"[Title/Abstract] OR "Adverse Events, Immune-Related"[Title/Abstract] OR "Adverse Event, Immune-Related"[Title/Abstract] OR "irAEs"[Title/Abstract] OR "Adverse event"[Title/Abstract] | 58846 |
| #5 | Lung Neoplasms[MeSH] | 307379 |
| #6 | "Pulmonary Neoplasms"[Title/Abstract] OR "Neoplasms, Lung"[Title/Abstract] OR "Lung Neoplasm"[Title/Abstract] OR "Neoplasm, Lung"[Title/Abstract] OR "Neoplasms, Pulmonary"[Title/Abstract] OR "Neoplasm, Pulmonary"[Title/Abstract] OR "Pulmonary Neoplasm"[Title/Abstract] OR "Lung Cancer"[Title/Abstract] OR "Cancer, Lung"[Title/Abstract] OR "Cancers, Lung"[Title/Abstract] OR "Lung Cancers"[Title/Abstract] OR "Pulmonary Cancer"[Title/Abstract] OR "Cancer, Pulmonary"[Title/Abstract] OR "Cancers, Pulmonary"[Title/Abstract] OR "Pulmonary Cancers"[Title/Abstract] OR "Cancer of the Lung"[Title/Abstract] OR "Cancer of Lung"[Title/Abstract] | 257254 |
| #7 | #5 OR #6 | 396492 |
| #8 | ("2016/01/01"[Date - Completion] : "2025/12/31"[Date - Completion]) | 10870634 |
| #9 | #3 AND #4 AND #7 AND #8 | 1227 |
